# Validation of a simultaneous PET/MR system model for PET simulation using GATE

**DOI:** 10.1186/2197-7364-2-S1-A45

**Published:** 2015-05-18

**Authors:** Florian Monnier, Hadi Fayad, Julien Bert, Holger Schmidt, Dimitris Visvikis

**Affiliations:** LaTIM UMR 1101, Brest, France; University Hospital of Tübingen, Tübingen, Germany

Simultaneous PET/MR acquisition shows promise in a range of applications. Simulation using GATE is an essential tool that allows obtaining the ground truth for such acquisitions and therefore helping in the development and the validation of innovative processing methods such as PET image reconstruction, attenuation correction and motion correction. The purpose of this work is to validate the GATE simulation of the Siemens Biograph mMR PET/MR system. A model of the Siemens Biograph mMR was developed. This model includes the geometry and spatial positioning of the crystals inside the scanner and the characteristics of the detection process. The accuracy of the model was tested by comparing, on a real physical phantom study, GATE simulated results to reconstructed PET images using measured results obtained from a Siemens Biograph mMR system. The same parameters such as the acquisition time and phantom position inside the scanner were fixed for our simulations. List-mode outputs were recovered in both cases and reconstructed using the OPL-EM algorithm. Several parameters were used to compare the two reconstructed images such as profile comparison, signal-to-noise ratio and activity contrast analysis. Finally patient acquired MR images were segmented and used for the simulation of corresponding PET images. The simulated and acquired sets of reconstructed phantom images showed close emission values in regions of interest with relative differences lower than 5%. The scatter fraction was within a <3% agreement. Close matching of profiles and contrast indices were obtained between simulated and corresponding acquired PET images. Our results indicate that the GATE developed Biograph mMR model is accurate in comparison to the real scanner performance and can be used for evaluating innovative processing methods for applications in clinical PET/MR protocols.

